# The PERK-Dependent Molecular Mechanisms as a Novel Therapeutic Target for Neurodegenerative Diseases

**DOI:** 10.3390/ijms21062108

**Published:** 2020-03-19

**Authors:** Wioletta Rozpędek-Kamińska, Natalia Siwecka, Adam Wawrzynkiewicz, Radosław Wojtczak, Dariusz Pytel, J. Alan Diehl, Ireneusz Majsterek

**Affiliations:** 1 Department of Clinical Chemistry and Biochemistry, Medical University of Lodz, 90-419 Lodz, Poland; wioletta.rozpedek@umed.lodz.pl (W.R.-K.); natalia.siwecka@stud.umed.lodz.pl (N.S.); adam.wawrzynkiewicz@stud.umed.lodz.pl (A.W.); radoslaw.wojtczak@p.lodz.pl (R.W.); 2 Department of Biochemistry and Molecular Biology, Hollings Cancer Center, Medical University of South Carolina, Charleston, SC 29425, USA; pytel@musc.edu (D.P.); jad283@case.edu (J.A.D.)

**Keywords:** neurodegenerative diseases, neurodegeneration, Alzheimer’s disease, Parkinson’s disease, PERK, endoplasmic reticulum stress, unfolded protein response, apoptosis, PERK inhibitors

## Abstract

Higher prevalence of neurodegenerative diseases is strictly connected with progressive aging of the world population. Interestingly, a broad range of age-related, neurodegenerative diseases is characterized by a common pathological mechanism—accumulation of misfolded and unfolded proteins within the cells. Under certain circumstances, such protein aggregates may evoke endoplasmic reticulum (ER) stress conditions and subsequent activation of the unfolded protein response (UPR) signaling pathways via the protein kinase RNA-like endoplasmic reticulum kinase (PERK)-dependent manner. Under mild to moderate ER stress, UPR has a pro-adaptive role. However, severe or long-termed ER stress conditions directly evoke shift of the UPR toward its pro-apoptotic branch, which is considered to be a possible cause of neurodegeneration. To this day, there is no effective cure for Alzheimer’s disease (AD), Parkinson’s disease (PD), Huntington’s disease (HD), or prion disease. Currently available treatment approaches for these diseases are only symptomatic and cannot affect the disease progression. Treatment strategies, currently under detailed research, include inhibition of the PERK-dependent UPR signaling branches. The newest data have reported that the use of small-molecule inhibitors of the PERK-mediated signaling branches may contribute to the development of a novel, ground-breaking therapeutic approach for neurodegeneration. In this review, we critically describe all the aspects associated with such targeted therapy against neurodegenerative proteopathies.

## 1. Introduction

Neurodegeneration is generally defined as progressive, irreversible loss of neurons, which may affect either the peripheral or central nervous system (CNS). There are numerous risk factors for neurodegenerative diseases, which include genetic polymorphisms, gender, poor education, oxidative stress, hypertension, inflammation, depression, stroke, tumors, diabetes, vitamin deficiency, immune and metabolic conditions, and frequent exposure to chemical agents, but among them the primary risk factor constitutes the increasing age of the world population [1]. Nowadays, almost every country in the world experiences a progressive increase in number of elderly people in their population. According to the global statistics by United Nations, in 2019 there were 703 million people aged 65 years or over, which has nearly doubled as compared to 1990, from 6% to 9% overall. It is also estimated that this number will rise further and that it will have doubled to 1.5 billion by 2050 (16%), which means that by this time one in six people will be aged 65 years and above. Currently, individuals 80 years and older are the fastest growing segment of the worldwide population [2].

Dementia, a clinical syndrome intrinsically linked to both aging and major neurodegenerative diseases, is an overall term that refers to loss of mental abilities. It covers a broad range of symptoms strictly associated with a significant decline in memory and aggravation of cognitive function [3]. Globally, 1–6% of the human population over 65 years old and 10–20% of those over 80 years old suffer from dementia [4]. The newest data have reported that due to the rapid aging of the world population, the incidence of dementia may significantly increase to approximately 65 million before 2030 and 115 million before 2050 [5]. Dementia constitutes a serious public health problem, as every four seconds one new case of dementia is diagnosed, resulting in 7.7 million cases per year [6].

Most of the neurodegenerative diseases, including Alzheimer’s disease (AD), Parkinson’s disease (PD), Huntington’s disease (HD), and prion disease, are closely correlated with the aggregation of the pathological proteins within the nervous tissue. This event constitutes the main hallmark of the neurodegenerative processes at the molecular level. Protein aggregates comprise structures composed of pathological unfolded or misfolded proteins such as α-synuclein (α-syn) in PD, amyloid β (Aβ) or tau protein in AD, mutant huntingtin protein (mHtt) in HD, as well as prion protein (PrP) in transmissible prion encephalopathies [7,8].

The majority of currently used treatments against the aforementioned neurodegenerative disorders may only alleviate symptoms and slow down progression of the disease, whereas the newest experimental research, in in vitro and in vivo models, has demonstrated a promising opportunity of pathogenic therapy for neurodegenerative diseases based on deuterium-reinforced polyunsaturated fatty acid (D-PUFA) administration. It has been reported that oxidative damage resulting from increased oxidative stress constitutes a critical factor in the development and progression of neurodegenerative diseases, including AD, PD, HD, as well as renal diseases. Interestingly, oxidative damage accompanies all AD stages, and thereby it plays a key role in AD pathogenesis. D-PUFAs, in comparison with regular hydrogenated PUFAs (H-PUFAs), are prominently more resistant to the reactive oxygen species (ROS)-mediated chain reaction of lipid peroxidation (LPO). A study by Elharram et al. has demonstrated that, in an oxidative stress-dependent mouse model of age-related cognitive impairment with AD-like biochemical and structural pathologies with deletion of the *aldehyde dehydrogenease 2* gene (*Aldh2^-/-^),* treatment with D-PUFAs for 18 weeks evoked an approximate 55% decrease of F2-isoprostanes (F_2_-IsoPs) and 20–25% decrease of prostaglandin F_2α_ (PGF_2α_) in both the cortex and hippocampus, as compared to H-PUFA-treated *Aldh2^-/^*^-^ mice for 18 weeks. Hence, this aforementioned study indicated that D-PUFAs significantly reduce LPO brain products in an *Aldh2^-/-^* mouse model of sporadic AD. Moreover, a study by Elharram et al. has also shown that *Aldh2^-/-^* mice fed a Western-type diet enriched with D-PUFAs exhibited better results in cognitive/memory tests, significantly resetting results of the *Aldh2^-/-^* mice fed a diet enriched with D-PUFAs to that of wildtype mice fed a typical laboratory diet. Thereby, a study by Elharram et al. has indicated that D-PUFAs may constitute a novel treatment strategy against AD, resulting in significant reduction of LPO and cognitive/memory decline in AD individuals [9]. Another study by Raefsky et al. has reported that D-PUFAs may enter the brain and subsequently incorporate into membrane lipids directly leading to suppression of neuronal lipid peroxidation. It has been demonstrated that deuterium was incorporated into docosahexaenoic acid (DHA) and arachidonic acid (ARA) in the hippocampus and cerebral cortex in amyloid precursor protein (APP)/presenilin-1 (PS1) double mutant transgenic mice fed a D-PUFA diet, as well as the D-PUFA diet being strictly correlated with significant decrease in products of LPO, including both F_2_-IsoPs and F4-neuroprostanes, in brain tissue of the mouse model of AD. Furthermore, as compared to AD mice on a H-PUFA diet, in AD mice on a D-PUFA diet the levels of Aβ_40_ and Aβ_38_ in the hippocampus were markedly reduced, with a trend to decreased concentrations of Aβ_42_ [10]. Oxidative stress plays a crucial role not only in the pathogenesis of AD, but also PD, as there is ample evidence that PUFA peroxidation products have been found in post-mortem tissue from PD individuals. Interestingly, study by Shchepinov et al. has reported that deuteration of bisallylic sites of PUFAs may trigger partial protection against nigrostriatal dopaminergic pathway failure in 1-methyl-4-phenyl-1,2,3,6-tetrahydropyridine (MPTP)-treated mice, which represent a mouse model of oxidative stress and cell death. It has been demonstrated that mice fed a H-PUFA diet, after MPTP exposure, exhibited a 78.7% loss of dopamine and a 64.3% loss of dopamine metabolite 3,4-dihydroxyphenylacetic acid (DOPAC), whereas, in the same experimental model, mice fed a H-PUFA diet exhibited only a 46.8% loss of dopaminergic (DA) and 36.5% loss of DOPAC. After MPTP treatment, D-PUFA-supplemented mice demonstrated higher levels of striatal tyrosine hydroxylase (TH) immunoreactivity than in H-PUFA-supplemented mice, which also indicates a neuroprotective role of D-PUFAs. Moreover, following MPTP treatment, a markedly higher number of nigral dopaminergic neurons have been indicated in the D-PUFA-supplemented mice in comparison with H-PUFA-supplemented mice. Thus, the above-mentioned research has demonstrated that deuteration of PUFAs may significantly slow oxidative cellular damage and constitute a modification of PD course [11]. As previously mentioned, oxidative damage also constitutes a key factor in the HD pathogenesis. Biomarkers of excessive LPO have been found both in mouse models of HD and HD individuals. Thereby, reduction of LPO may constitute a promising treatment strategy against HD. A study by Hatami et al. has demonstrated that Q140 knock-in (Q140 KI) mice, which represent a mouse model of HD, fed a diet enriched in D-PUFAs exhibited markedly decreased level of F_2_-IsoPs in the striatal tissue by approximately 80% as compared to H-PUFA-supplemented Q140 KI mice, suggesting that D-PUFAs relevantly decrease LPO in a mouse model of HD. Moreover, D-PUFA treatment mitigates cognitive impairment in Q140 KI mice, as after 5 months of consumption of a diet enriched in deuterium there was found to be significantly improved performance in novel object recognition tests, without changing motor deficits and accumulation of huntingtin protein (Htt). Hence, after further detailed investigations, D-PUFA administration may constitute a novel treatment strategy against HD [12].

Moreover, recent studies have indicated that pathogenesis of neurodegenerative diseases may be correlated not only with oxidative stress, but also with a significant alteration in protein homeostasis within the lumen of the endoplasmic reticulum (ER), which evokes the ER stress conditions. As the subsequent molecular event, the protein kinase RNA-like endoplasmic reticulum kinase (PERK)-dependent unfolded protein response (UPR) signaling pathways, which play a key role in memory and neurodegeneration, are directly activated [13]. Interestingly, the UPR is characterized by both a pro-adaptive and pro-apoptotic role. The newest data have indicated that under severe or long-term ER stress conditions, the pro-apoptotic branch of the UPR is activated, resulting in neuronal cell death. Thus, inhibition of the PERK-mediated, pro-apoptotic UPR signaling branches on the molecular level may also contribute to the development of a novel, targeted treatment strategy against neurodegeneration [14].

## 2. Alzheimer’s Disease

Among a broad range of neurodegenerative diseases, AD constitutes 60–70% of all types of dementia [15]. Of the world population, 26.6 million were affected by AD in 2006, but currently it is expected that the AD prevalence may quadruple by 2050; thus, 1 in 85 persons will suffer from AD [16]. Nowadays, the AD prevalence is approximately 5% in people aged over 65 years old, but it significantly increases up to 20% in the group of people over 85 years of age [5]. On the basis of the age of onset, AD is clinically divided into two types: early-onset AD (EOAD) and late-onset AD (LOAD). The Dementia Mutation Database indicates that approximately 300 mutations in presenilin-1 (PS1) and presenilin-2 (PS2) and 635 mutations in amyloid precursor protein (APP) constitute causative factors for EOAD development [17]. EOAD is characterized as developing in individuals before age 65, whereas LOAD shows symptoms in individuals after age 65. The majority of cases of familial AD (FAD) are strictly correlated with EOAD, and they account for only 5–10% of all AD cases. Thereby, sporadic cases of AD mostly present LOAD and constitute 90–95% of all AD cases [18].

AD is an age-related, progressive, neurodegenerative disease of the CNS, characterized by rapid progression of memory loss and significant impairment of multiple cognitive functions [19]. There are three general stages of AD: mild, moderate, and severe [20]. The time course of AD averages about 7–10 years. Impaired recent memory and other cognitive dysfunctions such as executive dysfunction, including concentration and problem solving abilities, constitute one of the first symptoms of AD. Progression of AD is strictly correlated with language and visuospatial impairment, as well as significant personality changes. The early stage of AD lasts 2–5 years, whilst the moderate stage 2–4 years and it finally leads to mental and physical disability and death at the late stage [21,22]. The main hallmarks of AD are extracellular deposition of senile plaques and intracellular aggregation of neurofibrillary tangles (NFTs) within the brain tissue [23]. The above-mentioned pathological aggregates constitute the primary biomarkers for AD diagnosis [24]. Senile plaques are mainly composed of the pathological form of Aβ, whereas the major component of NFTs is the hyperphosphorylated form of tau protein [25,26]. Numerous hypotheses regarding the AD etiology have been presented, but the AD pathogenesis still remains not fully understood [27]. Pathological changes within the brain tissue during the early stages of AD start in the entorhinal cortex and hippocampus. They are subsequently spread into the temporal, parietal, and frontal association cortices. At the early stages of AD, significant neurodegeneration occurs in poorly myelinated limbic neurons in the hippocampus and association cortex, directly related to memory and learning. Degeneration of highly myelinated neurons occurs only during the final phases of AD [28].

High content of PUFAs and transition metals within the brain tissue as well as high oxygen utilization and reduced antioxidant defense provide an environment susceptible to oxidative damage that is strictly correlated with the early stages of AD. ROS-mediated non-enzymatic LPO of PUFAs constitute a primary factor contributing to oxidative damage directly leading to altered membrane fluidity and significant changes in membrane-bound enzymes and receptors. ROS-initiated LPO of PUFAs begins by abstraction of bis-allylic hydrogens resulting in resonance-stabilized free radicals generation, which may react with molecular oxygen and finally form lipid peroxyls. Peroxyls abstract a hydrogen atom off an adjacent PUFA molecule, thus they play a crucial role in propagation of chain reaction. LPO leads to the creation of byproducts derived through either endoperoxide or hydroperoxide intermediates, decomposition of which results in creation of reactive aldehydes including among others: malondialdehyde, acrolein, 4-hydroxynonenal (HNE), and 4-hydroxyhexenal (HHE) [9]. Thereby, the progressive neurodegeneration process in AD is closely correlated not only with excessive deposition of Aβ, but also with oxidative stress and disruption of Ca^2+^ homeostasis, which subsequently trigger hyperexcitability and excitotoxicity of the neuronal network. It has been demonstrated that brain tissue is characterized by a high content of PUFAs such as ARA, DHA, and eicosapentaenoic acid (EPA) that are produced from linoleic acid and linolenic acid, which are susceptible to ROS attack directly leading to LPO. The above-mentioned molecular event results in significant membrane PUFA damage; generation of F2, F3, and F4 isoprostanes; and highly reactive HNE and HHE. There is ample evidence that oxidative stress and LPO play a crucial role in synaptic dysfunction and neurodegeneration in AD. It has been revealed that HNE evokes disruption of Ca^2+^ homeostasis and energy metabolism, directly leading to neuronal apoptosis in AD course [10]. 

Aggregation of misfolded and unfolded pathological proteins, occurrence of oxidative stress, and multiple metabolic disturbances inside the neurons of brain tissue represent the characteristic hallmarks of AD [29]. Currently, due to insufficient treatment strategies against AD, which are only symptomatic, novel therapeutic strategies based on inhibition of PERK-dependent signaling branches are under detailed investigation [30].

The main hallmark of AD brains constitutes deposition of insoluble senile plaques mainly composed of a longer, pathological form of Aβ composed of 42 amino acids (Aβ_42_) [31]. There is ample evidence that level of Aβ_42_ is significantly increased in AD brains due to its elevated hydrophobicity, which may facilitate its oligomerization and subsequent deposition in the form of toxic senile plaques among neurons of AD brain tissue [32]. Aβ is directly generated via proteolytic cleavage of the APP by three secretases: α, β, and γ [33]. APP constitutes a transmembrane protein, expression of which occurs in multiple cell types including neurons [34]. The *APP* gene on chromosome 21 has three most common isoforms: *APP695*, *APP751*, and *APP770*, whereas only the *APP695* is predominantly expressed in the CNS [35]. It has been reported that there are 32 *APP*, 179 *PS1*, and 14 *PS2* gene mutations directly leading to deposition of senile plaques within the brain tissue, thus development of autosomal dominant EOAD [35]. APP mutations at the cleavage sites for APP processing via secretases β and γ result in increased Aβ generation into the luminal/extracellular compartment [36]. PS1 and PS2 act as a component of γ-secretase catalytic subunit [37]. There is a plethora of in vitro and in vivo studies, which have demonstrated that PS1 and PS2 mutations markedly increase generation of Aβ and the ratio of Aβ_42_ to Aβ_40_ [38].

APP processing occurs through two distinct pathways: non-amyloidogenic or amyloidogenic [39]. Non-amyloidogenic APP processing occurs under physiological conditions with activation of α and γ secretases [40]. α-Secretase, to prevent Aβ generation, cleaves APP between Lys^16^ and Leu^17^ within the Aβ sequence, resulting in generation of soluble APPs_α_ and membrane-bound C83 fragments. Afterwards, C83 is cleaved in the γ-secretase-dependent manner into the APP intracellular domain (AICD) and p3 fragments. Function of p3 still remains unclear, whereas AICD may create a complex with several factors including stabilizing factor Fe65 and act as a transcription factor of a broad range of genes, including *neprilysin (NEP)*, which enhances Aβ degradation [41]. Alternatively, under pathological conditions, in the APP cleavage via the amyloidogenic pathway the central role is played by β and γ secretases. β-Secretase cleaves APP at the Asp^1^ site, which releases two products: soluble ectodomain APPs_β_ and C-terminal fragment C99. C99 is subsequently cleaved by γ-secretase into AICD and 39–42 amino acid Aβ, which promote formation of senile plaques among neurons of brain tissue [42]. γ-Secretase-mediated processing of C99 generates both Aβ_42_ and Aβ_40_, which may constitute a components of neuritic plaques, but Aβ_42_, as compared to Aβ_40_, is characterized by higher neurotoxicity and aggregation ability [43].

In the pathogenesis of AD, enhanced expression of β-secretase, the major factor that promotes accumulation of pathological senile plaques within the nervous tissue, directly leads to neuronal loss, synaptic failure, and significant reduction of brain volume, resulting in memory deficits and impairment of cognitive function. Several studies have confirmed the overexpression of *BACE1*, encoding for β-secretase or β-site amyloid precursor protein cleaving enzyme 1 (BACE1), in brain tissue samples obtained from AD patients, together with other hallmarks of AD pathology [44,45,46]. Numerous data have indicated a significantly elevated level of β-secretase in post-mortem AD brains. Moreover, overexpression of both β-secretase and phosphorylated form of the translation initiation factor 2α (p-eIF2α), which constitutes the main substrate of PERK, was observed in mouse model of amyloid deposition expressed five familial AD (FAD) mutations (5XFAD) [47]. Both phosphorylated form of PERK (p-PERK) and p-eIF2α levels were markedly increased in hippocampal neurons of AD brains [48]. Moreover, Stutzbach et al. found elevated levels of p-PERK and p-eIF2α in the hippocampal pyramidal cells and in the frontal cortex [49]. Interestingly, overactivation of cytosine-cytosine-adenosine-adenosine-thymidine (CCAAT)/-enhancer-binding protein homologous protein (CHOP), another PERK downstream target, has been recently found to be strictly associated with the occurrence of all hallmarks of AD. Upregulation of CHOP resulted in the following sequence of events: generation of ROS, oxidative damage, high level of Aβ, perturbation in iron homeostasis, neuroinflammation, and eventually neuronal cell death via apoptosis [50].

However, on the basis of data from the other sources, the involvement of PERK-dependent UPR signaling pathway in the process of both AD-related amyloidogenesis and tauopathy is still debatable. According to the study by Sadleir et al., 5XFAD transgenic mice with the enhanced expression of *APP* and *PS1* do not exhibit activation of ER stress conditions nor activation of UPR signaling. The levels of UPR-related proteins such as chaperone binding immunoglobulin protein (BiP), phosphorylated inositol-requiring enzyme 1 (p-IRE1α), p-eIF2α, activating transcription factor 4 (ATF4), or CHOP in AD mouse model were not significantly elevated as compared to the non-transgenic control mice. Interestingly, the experiment has also been performed in 5XFAD mice with silenced *BACE1* expression with similar results. These results imply that ER stress might not be the specific hallmark of AD, or at least is not induced by overexpression of *APP* and *PS1* [51]. A separate study by Spatara et al. has proven that the activation of the UPR signaling pathway is absent in several models of tauopathies such as the SH-SY5Y cell line, 4R tau- and P301L tau-expressing HEK293 cells, or PS19 transgenic mice [52]. These theory is supported by another piece of evidence that was provided by Pitera et al. In their study, no activation of UPR was reported in a rTg4510 mouse model that shared the expression of mutant tauP301L as well as in the primary hippocampal neurons of E15–E18 C57Bl/6 mice [53].

Currently used treatment modalities are symptomatic and may only temporarily alleviate problems with memory and cognitive function. Furthermore, the clinical effect of currently used treatments against AD is modest, thereby development of novel, targeted treatment strategies may solve problems connected with AD treatment. For instance, the newest data have reported that fine-tuning of the eIF2α phosphorylation may play a key role in controlling mnemonic processing. It has been demonstrated that a decreased level of the eIF2α phosphorylation directly evokes enhanced long-term memory consolidation and synaptic plasticity, whereas enhanced phosphorylation of the eIF2α causes their impairments. Thereby, genetic and pharmacological manipulations of the eIF2α phosphorylation on learning and memory processes are currently under detailed investigation [54].

## 3. Parkinson’s Disease

PD constitutes the second most common neurodegenerative disease after AD, with over 6 million people affected worldwide in 2016, but it is considered the fastest growing in terms of prevalence, disability, and mortality [55]. The number of individuals suffering from PD more than doubled from 1990 to 2016, and this amount is projected to progressively increase up to 9 million by 2030 [56] due to the aging society and high sociodemographic index [55]. PD is generally characterized by two major hallmarks: selective loss of dopaminergic (DA) neurons in substantia nigra pars compacta (SNpc) and accumulation of α-syn aggregates called Lewy bodies, which are sufficient to confirm the diagnosis in idiopathic forms of the disease [57]. However, the actual cause of neurodegeneration in PD still remains unclear due to its complexity, and thus it needs to be further elucidated. Nowadays, the pathogenesis of PD is considered multifactorial and connected with proteasome inhibition, impaired vesicle trafficking, alterations in calcium regulation, and mitochondria and lysosome dysfunction, all of which trigger oxidative stress within DA neurons [58,59].

PD is certainly age-related, as its incidence sharply increases with age and it becomes even 5- to 10-fold higher among individuals over the age of 60 [57]. Currently, the disease onset is estimated between 65 to 70 years of age, whereas its morbidity rates to a peak around age of 85–89 [60]. The other major risk factors may be divided into endogenous, which include, among others, gene mutations or history of melanoma, as well as exogenous, such as toxic exposure, traumatic brain injuries (TBI), and some dietary habits [61]. Overall, there have been 26 genes implicated in PD pathogenesis identified, although it is considered that environmental factors are more common causes of PD onset [61,62]. Among genes most frequently associated with PD, *leucine-rich repeat kinase 2 (LRRK2), synuclein alpha (SNCA), vacuolar protein sorting-associated protein 35 (VPS35),* and *glucosylceramidase beta (GBA)* can be indicated, mutations of which occur in autosomal dominant PD (ADPD), as well as *parkin RBR E3 ubiquitin protein ligase (Parkin), pten-induced putative kinase 1 (PINK1),* and *protein deglycase (DJ1)*, linked to early-onset autosomal recessive PD (ARPD) [57,62]. In general, *SNCA* encodes for α-syn, whereas the other above-mentioned genes are involved in autophagy, mitochondria metabolism, proteostasis, and chaperoning, all of which are deregulated in PD [63,64]. On the contrary, metabolic products of a drug called MPTP, as well as rotenone and some other pesticides, were confirmed to cause an acquired form of PD via interfering with the redox balance within midbrain neurons [65,66]. Both TBI and mild traumatic brain injury (MTBI) may contribute to development of PD due to disruption of the blood–brain barrier (BBB), subsequent induction of neuroinflammation, glutamate release, mitochondrial dysfunction, and eventually accumulation of α-syn [67,68].

Bradykinesia, resting tremor, postural instability, and muscular rigidity represent the specific symptoms of PD, which are connected with degeneration of DA neurons and thus result in neurotransmission failure within the nigrostriatal pathway [64,66,69]. Due to neural plasticity, it is estimated that a significant amount of DA neurons (approximately 30%) must have already been damaged by the time of the occurrence of the symptoms [66,70,71]. Other common extrapyramidal motor disturbances that have been noted among PD patients include muscle stiffness, motor blocks (freezing), akinesia, mask-like face expression, flexed posture, irregular arm swing, abnormal gait, and balance impairment [72,73]. Autonomic function may also be disturbed and it is commonly manifested by gastrointestinal motility disorders (e.g., constipation), urinary incontinence, orthostatic hypotension, or sexual dysfunction [64,72,74,75]. Symptoms such as cognitive decline, anxiety, depression, and other behavioral or sleep disorders are also frequently observed in PD sufferers [64,76,77]. In general, non-motor features usually occur many years prior to the motor symptoms’ onset [64,77], among which the olfactory loss (hyposmia or anosmia) is regarded as the most relevant early sign of PD, although being frequently overlooked [78]. All of the above-mentioned symptoms worsen with age, significantly decrease the quality of life in patients by impairing their daily functioning, and eventually lead to permanent disability.

Although many therapeutic options are available for PD treatment nowadays, none of them are considered flawless, nor do they provide full recovery. In most cases, despite alleviation of the symptoms, the progression of the disease cannot be affected. Moreover, in view of the recently reported drug resistance, it becomes even more complicated to deter the disease progression. 

The neurons of the substantia nigra (SN) region are, among other cell types, particularly susceptible to metabolic and oxidative stress. Indeed, a study by Floor et al. found that SN contained twice as many oxidized proteins as other regions of post-mortem brains in healthy individuals [79]. This is not surprising in light of the fact that SN neurons possess particularly long, unmyelinated axons; numerous synapses; as well as exhibit autonomous, altered calcium metabolism, all of which require substantial energy [66]. On the other hand, it has been confirmed that DA neurons have decreased levels of two major antioxidant defense agents: ferritin, which links iron ions and stores them in a non-toxic form, and glutathione, the main ROS scavenger. Conversely, they contain increased amounts of neuromelanin pigment, free iron ions, and dopamine with its metabolites, all of which are capable of induction of ROS [65,80]. 

α-Syn is a small (14 kDA, 140 amino acids), soluble protein encoded by the *SNCA* gene [66,81]. To date, multiplications of *SNCA* as well as six missense mutations (particularly A30P, A53T, and E46K) have been identified in familial forms of PD [66,82]. It has been found that α-syn levels correlate with PD onset, as duplication of *SNCA* resulted in late-onset ADPD, whereas triplication resulted in early onset PD [83]. The presence of α-syn is observed, apart from in PD, in a wide spectrum of other neurodegenerative disorders, such as dementia with Lewy bodies (DLB), PD with dementia (PDD), pure autonomic failure (PAF), or multiple system atrophy (MSA), commonly referred to as alpha-synucleinopathies [65,84]. α-Syn is abundantly expressed in the brain (estimated at about 1% of all cytosolic proteins), where it is localized in presynaptic membranes and vessicles to maintain neurotransmission and synaptic plasticity [74,82,84]. Moreover, expression of *SNCA* has recently been associated with iron and redox metabolism, both on transcriptional and post-transcriptional level [65].

Monomers and tetramers constitute the physiological forms of α-syn, whereas oligomers and fibrils constitute the pathogenic forms [66], among which the oligomeric species are considered the most harmful [85]. Structurally, the α-syn monomer contains three structural regions: the N-terminal domain (1–60) with a multi-repeated consensus sequence (KTKEGV), the central domain (61–95) or the non-amyloid-beta component (NAC) with highly hydrophobic motif, and the C-terminal domain (96–140) with acidic proline residues [64,69,81]. The central NAC domain, highly amyloidogenic, is normally protected by charges of N- and C-terminal regions, thus perturbations of this interaction (e.g., in case of mutations) may contribute to α-syn aggregation [86]. α-Syn monomers may undergo conformational changes and self-assemble into oligomeric species under appropriate conditions, such as the above-mentioned mutations, oxidative stress caused by 4-hyroxy-2-nonenal, cytochrome c or H_2_O_2_ release, as well as post-translational modifications, which cause the change of conformation from α-helix to β-sheet [72,74,85]. Misfolded α-syn becomes in turn progressively insoluble and accumulates in the form of intracellular inclusions in neuronal somas called Lewy bodies (LBs) or dendrites and axons as Lewy neurites (LNs) [70,87]. LBs are highly organized, spherical aggregates composed of insoluble eosinophilic amyloid surrounded by ubiquitinated α-syn fibrils abnormally phosphorylated at Ser129 [64,88]. Accumulation of LBs induces widespread toxicity within various cellular organelles, impairs numerous cellular functions such as formation of soluble NSF attachment protein receptor (SNARE) complex, dopamine neurotransmission and synaptic-vesicle motility, and eventually leads to neurodegeneration of DA neurons [64,89]. The decline in proteolytic defense mechanisms, which is intrinsically connected with increasing age, causes disturbances in autophagy–lysosomal and ubiquitin–proteasome systems, both of which are involved in maintenance of α-syn proteostasis [57]. In effect, the mutant forms of α-syn cannot be efficiently degraded by protein clearance mechanisms, and hence they disrupt loading and clearance of the other cargoes, resulting in a vicious cycle [72]. Interestingly, both increased levels of wild-type α-syn and presence of its abnormal forms have been proven to accelerate the accumulation process [64]. Such α-syn aggregates subsequently propagate through interconnected circuits of the brain in a prion-like mechanism, and the spreading closely correlates with the disease progression [82].

Numerous data have demonstrated that oxidative stress plays a crucial role in PD pathogenesis. Oxidized PUFAs may evoke a disruption of cellular homeostasis via oxidation of DNA, proteins, and other cellular targets such as α-syn and dopamine. PUFAs constitute a key component of lipid membranes as well as early targets of ROS-mediated oxidation, which negatively affects membrane properties such as fluidity. Moreover, oxidized PUFAs may also damage DNA and proteins by HNE and HHE, malondialdehyde (MDA), and acrolein. It has also been reported that LPO within mitochondria is closely connected with apoptosis. Cardiolipin oxidation constitutes a prominent factor responsible for apoptosis initiation via cytochrome c release and finally activation of proteolytic cascade leading to cell death by apoptosis and subsequently PD pathogenesis [11]. 

Initially, the disruptions in protein clearance within the ER lumen trigger activation of a pro-adaptive branch of the UPR signaling pathway, the main aim of which is to restore cellular proteostasis. Although α-syn has not been identified as a resident protein of the ER, a plethora of studies have proven that it directly interacts with both ER and vesicular traffic components and thus induces ER stress conditions [85]. It has been reported that α-syn impairs vesicular trafficking from the ER to the Golgi apparatus via direct interaction with the ras-associated binding 1 (RAB1) GTPase [90,91] or activating transcription factor 6 (ATF6), one of the main UPR signaling branches. α-Syn inhibits processing of ATF6 via coat protein complex II (COPII)-mediated ER–Golgi transit induced upon ER stress, which in turn impairs ATF6 activation, attenuates its cytoprotective effect, and triggers apoptosis [92]. Moreover, a study by Betzer et al. demonstrated that α-syn aggregates may interfere with calcium metabolism in neurons via activation of ER calcium pump SERCA, which as a result sensitizes cells to ROS production and to apoptosis [93]. Another recent study by Paiva et al. reported that A30P α-syn upregulates the expression of the *collagen type IV alpha 1 chain (COL42A)* gene encoding for collagen IV, an important secretory cargo within the Golgi apparatus, resulting in altered ER/Golgi morphology and increased susceptibility to ER stress in DA neurons [94]. Importantly, numerous studies have confirmed the direct interaction between α-syn aggregates and GRP78 chaperone, which may directly activate the UPR signaling pathway when it binds to misfolded proteins [89,95,96].

## 4. Other Neurodegenerative Diseases

HD is a pure genetic, late-onset autosomal dominant disease, in contrast to other neurodegenerative disorders, which in most cases are sporadic (up to 90%) [73]. The prevalence of HD is estimated at 5–8/100.000 and it usually affects people at a mean age of 35, regardless of gender [87]. The disease remains progressive and incurable as well as finally leads to imminent death approximately 15 years after the diagnosis [97,98]. Characteristic symptoms of HD include involuntary, jerky movements of the limbs named twitching or chorea, as well as dystonia, bradykinesia, myoclonus, dementia, personality changes, and depression [73,99]. The onset of psychiatric and cognitive defects appears earlier than that of motor abnormalities, during early to mid-adult life [98,100]. All mentioned symptoms are caused by a premature loss of the gamma-aminobutyric acid (GABA)-ergic medium-sized spiny neurons of the striatum, projecting to SN pars reticulata and pallidum, as well as other brain regions such as the basal ganglia, cortex, thalamus, or hypothalamus, although to a lesser extent [87,101,102]. It has been reported that significant atrophy of the caudate and putamen occurs years before manifestation of the symptoms [101]. HD is caused by an expansion of cytosine-adenine-guanine (CAG) repeats in the 5’ coding region of the *HTT* gene encoding for a polyglutamine (polyQ) stretch near the N-terminal region of Htt [100,103,104]. The number of CAG trinucleotide repeats is highly polymorphic and differs between healthy individuals from 11 to 34 glutamine repeats, but in HD it exceeds the pathological threshold of 35 repeats [87,103]. Interestingly, the increasing number of expanded CAG repeats accelerates the neurodegeneration process and inversely correlates with the age of both disease onset and death [73,104]. Other “polyglutamine disorders” characterized by polyQ repeats that trigger region-specific neurodegeneration include Machado–Joseph disease (MJD), spinocerebellar ataxia (SCA), or spinobulbar muscular atrophy (SBMA) [100]. Mutation in the *HTT* gene is completely penetrant and the expansion of 40 or more CAG repeats within the Htt leads to toxic gain-of-function and progressive accumulation of misfolded mHtt as intracellular oligomers called inclusion bodies (IBs) [87,97]. Although both soluble and aggregated forms of mHtt induce cytotoxicity, soluble/oligomeric mHtt are regarded as more toxic [104]. It has also been observed, as in the case of α-syn, that such Htt inclusions often co-localize with ubiquitin [98]. IBs aggregate within the cytoplasm and the nucleus and eventually contribute to the selective loss of striatal neurons [87] via induction of series of pathological events, which include transcriptional dysfunction, mitochondrial disturbances, vesicular transport impairment, ER stress, perturbation of calcium signaling, protein trafficking and amino acid metabolism, proteasome inhibition, as well as apoptosis [97]. Although mutant Htt has not been found to directly accumulate inside the ER lumen, it has been confirmed to interact with the ER membrane and thus interfere with ER function indirectly. Several models have been suggested to explain this phenomenon regarding excitotoxicity, oxidative stress, mitochondrial and proteasome dysfunction, transcriptional disturbances, alterations in axonal transport, or in the secretory pathway [98]. Recent reports have implied that Htt may cause an early defect of the endoplasmic-reticulum-associated protein degradation (ERAD), as it impairs the flux of proteins targeted for degradation, and in turn fosters the accumulation of misfolded proteins within the ER. Mechanistically, mHtt fragments entrap ERAD-associated proteins, namely, glycoprotein 78 (Gp78), nuclear protein localization protein 4 (Npl4), ubiquitin fusion degradation protein 1 (Ufd1), and protein 97 (p97), and thus inhibit protein trafficking. The IBs have also been found to impair the protein degradation process via inhibition of the ubiquitin–proteasome system (UPS) [105]. Nevertheless, the actual link between HT and ER stress is yet to be elucidated.

Prion diseases are rare, fatal, and transmissible neurodegenerative disorders that may affect both humans and animals [106]. They may be either inheritable or infectious and include inter alia, Creutzfeldt–Jakob disease (CJD), Gerstmann–Sträussler–Scheinker syndrome (GSS), fatal familial insomnia (FFI), and Kuru [107,108,109]. The clinical manifestation of prion disease varies considerably according to the type of disorder and thus the affected brain region, but it is usually associated with severe neurological, psychiatric, and cognitive abnormalities [108]. Familial forms of CJD, GSS, or FFI are caused by mutations or insertions in the *prion protein (PRNP)* gene, encoding for cellular prion protein (PrPc) which naturally occurs in the organism (PrP^c^) [73]. Sporadic forms, on the other hand, may be caused by spontaneous misfolding of PrP^c^ or rare somatic mutations in *PRNP* [108]. PrP^c^ is a soluble, 209-amino acid, α-helix-rich protein, with the sequence capable of induction of apoptosis (residues 106–126) and highly conserved hydrophobic C-terminal and N-terminal regions, which anchor the protein into the ER lumen [108]. The role of PrP^c^ has not been fully elucidated, although it has been linked to neuronal growth and survival, cytoprotection against cellular stress, and tumor necrosis factor (TNF)- and BCL2 associated X (BAX)-mediated cell death [74]. Pathologically, the disease is caused by abnormal folding of PrP^c^, resulting in the development of abnormal, protease-resistant scrapie isoform (PrP^Sc^) [106,110]. In comparison with its native form, PrP^Sc^ is characterized by three-dimensional conformation and higher β-sheet structure. It is prone to aggregate in the form of diffuse synaptic plaques, together with amyloid, in the brain and lympho-reticular tissues, leading to spongiform encephalopathy, progressing neuronal loss, astrocytosis, and microgliosis [74,107]. Of note is that the interaction of PrP^Sc^ with PrP^c^ induces abnormal folding of the native form, which in turn becomes infectious, further amplifies the protein misfolding, and propagates from one cell to another [74]. This event has been linked to aging, as a consequence of failure in antioxidant defense mechanisms, decrease in chaperone activity, and impaired protein degradation process [73]. Moreover, it has been proven that PrP^Sc^ is able to self-replicate, despite the absence of genetic material [111]. 

The ability of misfolded proteins, other than prion protein, to constitute a template for the seed creation from the physiologic form of the same kind of protein, propagate between cells, and spread across the anatomical pathways, is commonly termed as a “prion-like property”. Interestingly, it has been reported that propagation of other misfolded proteins in non-infectious neurodegenerative diseases may occur by the mechanisms similar to these characteristic for prion pathogenesis. Aβ and tau proteins in AD; α-syn in PD; TAR DNA-binding protein 43 (TDP43), tau, or fused in sarcoma (FUS) proteins in frontotemporal dementia (FTD); and TDP43 in motor neuron disease exhibit some properties of the misfolded prion protein. Multiple pieces of in vitro and in vivo evidence have demonstrated that the self-propagation mechanism by seeding and aggregation of similar misfolded proteins constitutes a characteristic hallmark of not only PrP^Sc^ but also Aβ, tau, α-syn, and TDP43 proteins [112]. It has been demonstrated in an in vitro cellular model that intracellular oligomers may constitute the seeding unit. Aβ seeding nucleation may be initiated inside the APP-producing cells upon their exposure to FAD brain extract [113]. Furthermore, it has been reported that seeding potency of Aβ is the greatest at the beginning of the Aβ aggregation, as well as concurring with a transient increase of the Aβ_42_/Aβ_40_ ratio. Seeding potency decreases with increasing formation of senile plaques in the brain tissue [114]. Thereby, compounds administrated during the early stages of AD, which may interfere with Aβ seeding formation and block the disease progression before the senile plaques aggregate and can be detectable, may represent a novel treatment strategy against AD [112]. Moreover, a tau protein may also constitute a seeding unit such as PrP^Sc^. The aforementioned molecular event has been confirmed both in in vitro and in vivo models. It has been suggested that aggregation of tau is initiated via phosphorylated tau fractions characterized by high molecular weight, which were found to be present in the cerebrospinal fluid (CSF) of an AD mouse model or AD individuals [115]. It has also been reported that tau, similarly to prions, propagates numerous conformations from different sporadic tauopathies in vitro as well as initiates distinct pathologies in vivo in mouse models demonstrating human diversity of tauopathies Interestingly, the mentioned conformations of tau are transmissible and may be passaged both in in vitro and in vivo models [116,117,118,119]. The seeding activity has also been confirmed for α-syn. Brain homogenates from patients with multiple system atrophy (MSA) are responsible for neurodegeneration both in cell models and in transgenic mice [120]. Additionally, oligomer creation is closely associated with the early stages of TDP43 misfolding has been confirmed for various frontotemporal lobar degeneration with TDP-43 inclusions (FTLD-TDP) types as well as in individuals with diagnosed hippocampal sclerosis with TDP43 pathology [121,122,123]. The cell-based seeding assay has demonstrated that extracts derived from brains with sporadic and familial FTLD-TDP were characterized by a higher seeding potency in comparison with control brain extracts [124]. As mentioned previously, numerous experimental data have reported that prion-like mechanisms are currently established for Aβ, tau, α-syn, and TDP43 proteins, which constitute crucial components in inducing seeding and subsequently evoke neurotoxicity and neurodegeneration. Many experiments in in vivo models have confirmed transmission and propagation as well as seeding potencies of misfolded proteins, other than prions, which are strictly correlated with neurodegeneration [112]. Transmission of protopathic seeds, characteristic for prion disease, have been found in in vivo research including Aβ transmission in transgenic mice, mutant P301S tau transmission in transgenic mice and from human tauopathies into transgenic mice, α-synuclein transmission in transgenic mice using synthetic fibrils and human MSA brain extracts, and TDP43 transmission in transgenic mice using human brain-derived FTLD-TDP extracts [124,125,126,127]. Transmission between humans has been described in prion diseases, whereas that prion-like mechanisms has been also observed in individuals that received pituitary-derived GH and had died of prion disease. In these patients, frequent cerebral amyloid angiopathy (CAA) and parenchymal Aβ have been confirmed [128]. Aβ transmission has also been demonstrated in patients who received contaminated dural grafts and in the post-neurosurgical patients [129,130].

**Figure 1:**
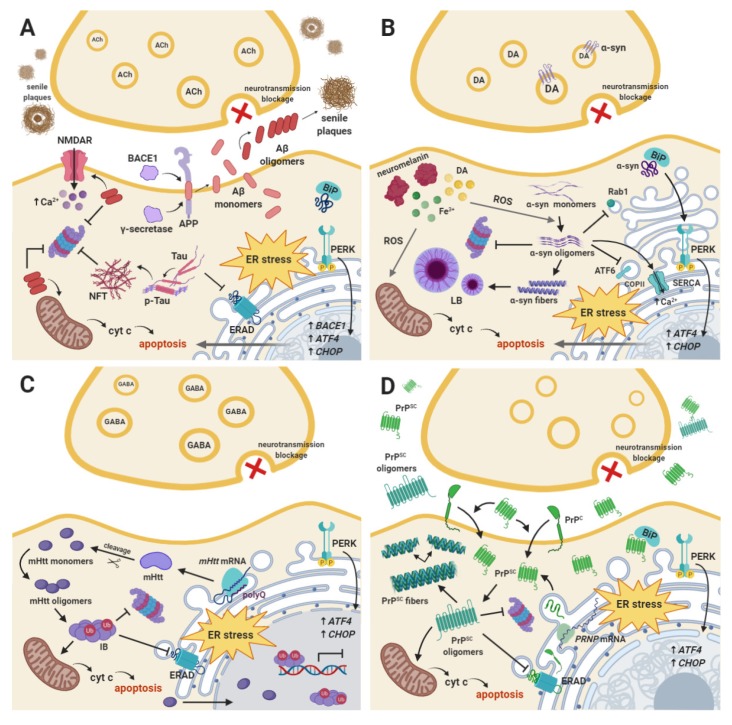
The schematic view of molecular perturbances resulting in subsequent activation of the protein kinase RNA-like endoplasmic reticulum kinase (PERK)-dependent unfolded protein response (UPR) signaling pathway in the following neurodegenerative disorders: (**A**) Alzheimer’s disease (AD), (**B**) Parkinson’s disease (PD), (**C**) Huntington’s disease (HD), and (**D**) prion disease. More details are included in the text of the manuscript.

PrP^Sc^ accumulation exhibits a deleterious effect in cells via different signaling cascades, particularly in the ER stress-mediated UPR signaling pathway [106]. PrP^Sc^ has been shown to accumulate within intracellular compartments such as the ER, disrupt Ca^2^^+^ homeostasis, and hence lead to ER stress-induced apoptosis. Several post-mortem studies have confirmed that UPR-related and apoptotic mediators are activated in infected cells [74], although the detection of this marker is difficult due to the long delay of autopsy in order to take infectivity precautions [107]. For instance, Moreno et al. have reported that accumulation of misfolded PrP resulted in increased phosphorylation of PERK and eIF2α that triggered attenuation of global protein synthesis within the brain tissue, and hence increased rate of synaptic failure and neuronal atrophy [131]. Conversely, several studies have denied the involvement of UPR in the pathogenesis of prion disease, suggesting that signs of UPR activation are less prominent in prion disease than in other types of neurodegenerative disorders such as AD. The presence of phosphorylated forms of PERK or eIF2α have not been found in post mortem samples of patients suffering from both genetic and infectious variants of CJD [132]. Likewise, neither p-PERK nor p-IRE1α, the second major branch of UPR signaling, have been detected in brains of scrapie-affected or CJD mice [109]. The main downstream target of the IRE1α branch of UPR, x-box binding protein 1 (XBP1), has also been excluded from involvement in prion pathogenesis, as reported by Hetz et al. [133]. On the other hand, aspects such as activation of caspase-12 in affected neurons as well as whether PrP^c^ binds to Aβ or α-syn oligomers still remain controversial, given that numerous different studies have demonstrated opposite results. However, there has been growing evidence for a cross-seeding process between misfolded proteins, as α-syn has been proven to be capable of inducing a transmissible spongiform encephalopathy with PrP accumulation [84] (Figure 1).

## 5. ER Stress and the UPR Signaling Branches

The ER constitutes the main cellular compartment for protein folding and assembly [134]. It is a highly complex organelle not only in its structure, but also in its function, as the ER plays a fundamental role in protein processing, which includes biosynthesis, folding, modification, and their transport. The ER is also an important organelle for biosynthesis and distribution of phospholipids and steroids, detoxification, energy metabolism, nucleus–cytosol signaling, and it is also responsible for the quality control of the newly synthesized proteins. Moreover, it plays a pivotal role in the maintenance of cellular calcium ion homeostasis [135,136]. Neuronal Ca^2+^ homeostasis and signaling are responsible for proper synaptic transmission, plasticity, and cell survival. Disturbances in Ca^2+^ homeostasis trigger significant dysregulation of neuronal physiology, leading to neuronal dysfunction and degeneration [137].

ER homeostasis is essential for cell survival, but a broad range of pathological conditions may disrupt that cellular state and evoke the ER stress conditions. They are directly activated upon glucose deprivation, redox imbalance, calcium homeostasis disruption, and as a response to the accumulation of improperly folded proteins within the ER lumen [29,138]. Aggregation of unfolded and misfolded proteins within the ER and subsequent ER stress conditions trigger activation of the UPR signaling branches, which are responsible for enhanced ER ability for protein folding and attenuation of global protein translation [139]. Interestingly, the UPR is characterized by a dual role, both pro-adaptive and pro-apoptotic. The major role of the UPR is the cellular adaptation for mild and moderate pathological conditions [140,141]. However, upon severe or long-termed ER stress conditions, or when the UPR is chemically or genetically impaired and the protein folding disruption is unable to mitigate, the pro-adaptive UPR signaling branches are insufficient to restore ER homeostasis, which consequently activates the pro-apoptotic arm of the UPR [139].

There are three major ER transmembrane proteins that act as sensors of ER stress conditions: PERK, IRE1, and ATF6 [142,143,144]. The aforementioned transmembrane ER transducers are directly activated by a common stimulus— the accumulation of pathological proteins within the ER lumen. The UPR induction is mediated via upregulation of a wide range of genes that are activated in an IRE1- and ATF6-dependent manner, as well as PERK-mediated inhibition of global protein synthesis and transcription of only selective sets of mRNAs [145]. The ER chaperone BiP, also known as 78 kDa glucose regulated protein (GRP78) or heat shock protein family A, member 5 (HSPA5), is commonly known as a major regulator of the ER homeostasis. BiP plays a fundamental role in folding and assembly of newly synthesized proteins and prevents their misfolding and aggregation. As a chaperone, BiP targets abrogated proteins for proteasome degradation and is responsible for the rapid initiation of the UPR signaling pathways [106]. Under ER homeostasis, BiPs bind to the lumenal domains of the ER transmembrane transducers such as PERK, ATF6, or IRE1 and maintain them in an inactive state. Increased level of misfolded and unfolded proteins within the ER lumen triggers dissociation of BiPs from PERK and IRE1 luminal domains resulting in oligomers formation and activation of the cytosolic effector domains of the sensors by trans-autophosphorylation. Thereby, dissociation of BiPs is necessary to activate PERK and/or IRE1 ER receptors [146]. ATF6, after BiP dissociation, is transported to the Golgi apparatus, where its two-step cleavage by the Golgi site-1 and site-2 proteases (S1P and S2P, respectively) occurs [147]. The main aim of the above-mentioned event is to generate the cytosolic domain that may be translocated into the nucleus, where it acts as a transcription factor for the UPR-specific genes [148].

Disturbances in all three signaling branches of the UPR are closely connected with the pathogenesis of a broad range of human diseases, but the PERK-mediated signaling pathways directly trigger attenuation of global protein translation that plays a major role in learning and memory processes, synaptic plasticity, and neuronal survival. Thus, there is a strong correlation between the dysregulation of the PERK-dependent branch of the UPR and the development and progression of neurodegenerative diseases [149].

### 5.1. PERK-Dependent pro-Adaptive Branch of the UPR

The plethora of studies have reported that the main aim of the UPR signaling pathways is to protect cells from adverse conditions due to pathological changes in the cellular environment. Moreover, UPR protects the organism by eliminating cells that were exposed to extreme and long-term ER stress conditions. However, the balance between the pro-adaptive and pro-apoptotic response upon activation of the ER stress conditions still remains unclear [150,151,152].

PERK belongs to the eIF2α kinase subfamily, which is composed of three additional members – the protein kinase C-related kinase (PKR), the general control nonderepressible 2 (GCN2), and the heme-regulated eIF2α kinase (HRI). All of them are activated via different stimuli: PKR by double-stranded viral RNA in infected cells, GCN2 by uncharged transfer RNAs (tRNAs) or UV irradiation, and HRI through hemin deprivation in erythroid cells [153]. PERK constitutes one of the three major ER transmembrane stress-responsive proteins. It is composed of two major parts, namely, C-terminal cytoplasmic domain with serine/threonine kinase activity and N-terminal ER lumenal domain, as well as the additional short hinge loop that links them. The N-lobe comprises three α-helices and five β-strands, whereas the C-lobe is composed of seven α-helices, two short β-strands, and a long activation loop [153,154].

Under homeostatic conditions in unstressed cells, PERK is presented as a homodimeric, transmembrane ER protein and remains inactive due to BiPs bound to its cytoplasmic domains. However, upon disturbances in the protein folding, resulting in activation of the ER stress conditions, BiPs dissociate from the cytoplasmic domains of PERK [155]. The aforementioned event triggers the transient conversion of PERK’s structure from a dimeric to a tetrameric state. After PERK tetramerization, activation loop of the first dimer may reach the catalytic site of the second dimer that directly leads to trans-autophosphorylation of the C-terminal PERK domain and subsequent phosphorylation of PERK substrates [154,156].

PERK activation promotes phosphorylation of the subunit α on Ser51 of the eIF2α, which acts as a major substrate of PERK [157,158,159]. This results in the attenuation of the global protein synthesis and, on the contrary, enhanced translation of only selective sets of mRNAs, including ATF4 [160]. Upon mild-to-moderate ER stress conditions, ATF4 constitutes a transcription factor for genes encoding proteins essential for restoration of cellular homeostasis, including ER chaperones, proteins required for proper amino acid metabolism, redox balance, autophagy, as well as cholesterol metabolism [135,161,162]. Thus, upon activation of the pro-adaptive UPR signaling pathway, transcription of genes encoding proteins such as BiP, calreticulin, calnexin, and protein disulfide isomerases (PDI) is significantly upregulated [163].

Another protein upregulated in a PERK-dependent manner is BACE1, which is directly involved in AD pathogenesis [160]. There is a body of evidence that an increased level of p-eIF2α is closely associated with higher expression of BACE1, which implies the involvement of the PERK-dependent UPR signaling pathway in BACE1 overexpression and promotion of amyloidogenesis. Numerous studies addressing this issue have been performed in vivo in a mouse model of AD with similar results [47,164,165,166]. However, to evaluate the role of eIF2α phosphorylation in Aβ-dependent BACE1 elevation in primary neuron culture and in the brain of the 5XFAD mouse model of aggressive amyloid pathology, Sadleir et al. used three genetic strategies: DNA damage-inducible 34 (GADD34) cytosine-adenine adeno-associated virus (CA-AAV) transduction, eIF2α S51A knock-in mutation, and *BACE1-yellow fluorescent protein (YFP)* transgene lacking upstream open reading frames (uORFs) in the 59 untranslated region (59UTR uORFs). Both in vitro and in vivo experimental models demonstrated that partial or complete reduction of eIF2α phosphorylation cannot inhibit the Aβ-mediated increased level of BACE1. Moreover, it has been shown that YFP-tagged BACE1, expressed from a transgene with a truncated BACE1 mRNA 59 UTR lacking uORFs required for p-eIF2α-mediated translational control, was increased and may aggregate around plaques in a pattern identical to endogenous BACE1 in an 5XFAD brain. Moreover, reduced level of p-eIF2α did not block Aβ-dependent APP elevation in primary neurons and also did not decrease Aβ levels or senile plaque pathology in 5XFAD brain. Thereby, aforementioned outcomes have demonstrated that, both in primary neurons and 5XFAD mice, eIF2α phosphorylation does not play a crucial role in Aβ-mediated BACE1 and APP elevation, as well as in amyloid pathology [167]. Hence, the above-mentioned findings suggest that the PERK-dependent elevation of BACE1 might be taken into consideration and further elucidated. 

There is ample evidence that activation of UPR signaling is present in the models of multiple neurodegenerative disorders. Markers of the UPR induction were found not only in brain tissues of animal models of neurodegeneration, but also in post mortem samplings of human brains with neurodegenerative disorders [149,168]. Thereby, activation of the UPR may be either somehow correlated with the pathogenesis of the diseases or could result from a completely different primary cause of neurodegeneration. As a protective signaling activated in response to different stressors, it may ultimately be unable to rescue remining neurons that are overwhelmed with prolonged stress conditions. Such a thesis should, however, be taken into consideration cautiously in light of the fact that numerous studies have declined the involvement of UPR in neurodegenerative processes.

### 5.2. PERK-Dependent pro-Apoptotic Branch of the UPR

Interestingly, failure of the pro-adaptive arm of the UPR occurs when the restoration of the cellular homeostasis is impossible. This event directly evokes activation of the pro-apoptotic branch of the UPR signaling pathway, but the underlying mechanisms responsible for the aforementioned molecular switch has not been completely elucidated [169]. There is ample evidence that, despite the pro-adaptive role of ATF4, it also has a negative impact on synaptic plasticity and memory consolidation [170,171,172,173]. ATF4, upon excessive or prolonged ER stress conditions, markedly upregulates expression of the *DNA damage-inducible transcript 3 (DDIT3)* gene that encodes CHOP, which represents the major pro-apoptotic factor of the UPR signaling pathways [174,175]. Under physiological conditions, cells are characterized by extremely low expression of CHOP. However, when the self-protect mechanisms within the cells are insufficient to overcome the ER stress, cells undergo CHOP-mediated apoptosis [50,176]. It has been reported that CHOP, as a transcription factor, may regulate expression of both pro- and anti- apoptotic genes, the products of which belong to the B cell lymphoma-2 (Bcl-2) family of proteins. Furthermore, CHOP may also upregulate expression of *DNA damage-inducible 34 (GADD34), Tribbles-related protein 3 (TRB3)*, and *endoplasmic reticulum oxidoreductin 1 (ERO1α)* [177].

Zinszer et al. have reported that, upon ER stress conditions in CHOP knock-out (KO) mouse embryonic fibroblasts (MEFs), cellular apoptosis was found to be significantly less frequent than in wild type MEF cells. Moreover, upon activation of ER stress in CHOP^+/+^ and CHOP^+/-^ mice, early expression of CHOP occurred, whereas in a CHOP^-/-^ animal model the evidence of cellular death was significantly reduced [178]. CHOP markedly enhances upregulation of genes encoding pro-apoptotic Bcl-2 homology 3 (BH3)-only proteins such as the p53 upregulated modulator of apoptosis (PUMA), Phorbol-12-myristate-13-acetate-induced protein 1 (NOXA), and Bcl-2-like protein 11 (BIM) [179]. These aforementioned pro-apoptotic BH3-only proteins play a key role in the mitochondrial-dependent cell death by apoptosis. PUMA, NOXA, and BIM promote activation of BAX and BCL2-antagonist/killer 1 (BAK), which subsequently oligomerize in the outer mitochondrial membrane (OMM) and create pores, resulting in the release of cytochrome c into the cell cytoplasm. This leads to formation of apoptosome and subsequent caspase activation, which directly evokes apoptosis [180]. Moreover, CHOP may also directly downregulate expression of anti-apoptotic Bcl-2 proteins [179,181,182]. ER stressors caused marked increase in PUMA and BIM level within mouse cortical neurons [183]. Due to deficiency in BIM and PUMA, apoptosis was significantly decreased in neuronal precursor cells in mouse hippocampus [184]. Moreover, under ER stress conditions, neuronal SH-SY5Y KO cells were characterized by decreased level of pro-apoptotic PUMA and BIM proteins [185]. Thus, consistent with the aforementioned data, the level of pro-apoptotic proteins such as BIM is markedly upregulated, as well as the level of anti-apoptotic proteins such as Bcl-2 being significantly decreased under ER stress conditions evoked by oligomeric Aβ treatment, which directly induces neuronal cell death [186].

Another target of CHOP constitutes TRB3, which due to pathogenesis of human diseases is characterized by a dual role. It has been reported that TRB3 plays an important role in a negative feedback loop under mild to moderate ER stress conditions, which may block its pro-apoptotic activity. Thus, the aforementioned TRB3-mediated molecular mechanism may result in restoration of cellular homeostasis. On the contrary, under adverse ER stress pathological conditions, TRB3 plays a critical role in Akt inhibition, resulting in apoptotic cell death [182].

Additionally, CHOP also enhances expression of growth arrest and DNA damage-inducible 34 (GADD34), which creates a complex with the catalytic subunit of protein phosphate 1 (PP1) that directly triggers dephosphorylation of eIF2α during later stages of the ER stress response [187]. Interestingly, p-eIF2α dephosphorylation plays a neuroprotective role, as it directly restores global protein translation [131]. It has been reported that expression of GADD34 is ER stress-dependent, as it was absent in PERK KO cells [188]. There is abundant evidence that increased level of GADD34 may significantly enhance apoptosis [189,190]. Mechanistically, p-eIF2α-mediated activation of ATF4/CHOP/GADD34 arm of the UPR signaling pathway inhibits the phosphorylation of eIF2α in a negative feedback loop and thus promotes translational recovery. This rapidly increases the level of ER nascent proteins entering the ER, enhances ER stress, and subsequently leads to cell death via apoptosis [191,192]. These findings are supported by the fact that salubrinal, a selective small-molecule inhibitor of eIF2α dephosphorylation, has been reported to prevent apoptotic cell death under prolonged ER stress conditions in the derived from a rat pheochromocytoma (PC12) cell line [193].

CHOP constitutes a transcription factor for the *ERO1α* gene, which plays a key role in the formation of disulfide bonds when cells remain under a homeostatic state. However, under severe, long-termed ER stress conditions, ERO1α evokes significantly elevated generation of H_2_O_2_, promoting a hyperoxidizing environment, and thus apoptotic cell death [194]. There is strong evidence that cerebral ischemia may constitute a significant risk factor for neurodegeneration [195,196,197]. It has been reported that cerebral ischemia evokes the ER stress conditions that activate the UPR signaling pathways [198]. In an animal model, global ischemia led to increased expression of CHOP and its downstream targets ERO1α in the hippocampal neurons [199]. CHOP-mediated upregulation of ERO1α causes activation of calcium-release channel inositol-1,4,5-trisphosphate receptor 1 (IP3R1), resulting in leakage of Ca^2+^ into the cell cytoplasm. Increased level of Ca^2+^ activates calcium/calmodulin-dependent protein kinase II (CaMKII), which in turn induces multiple apoptotic signaling pathways [200], including activation of nicotinamide adenine dinucleotide phosphate-oxidase (NADPH) subunit 2 (NOX2) and subsequently increases concentration of reactive oxygen species (ROS). This molecular event results in a positive feedback loop, as ROS generated via NADPH oxidase promote enhanced expression of CHOP, leading to apoptotic cell death [201,202] (Table 1).

## 6. Modulatory Compounds of the UPR Signaling Pathways

Recent data have reported that perturbations in the UPR signaling branches might play a role in the development and progression of multiple human diseases, including neurodegenerative disorders. It has been demonstrated that overactivation of PERK-eIF2α phosphorylation occurs in several transgenic mouse models of β-amyloidosis and tauopathy. There is also evidence that the PERK-eIF2α signaling pathway may potentially be involved in the progression of AD and other neurodegenerative diseases, the main hallmarks of which are memory deficits [54]. These data led to the prediction that pharmacological modulation of the PERK-dependent UPR signaling pathways may result in a significant neuroprotective effect. Thereby, targeting of the ER stress-dependent UPR signaling branches may contribute to the development of a novel, ground-braking treatment strategy against neurodegeneration [107,149,258].

### 6.1. GSK2606414

GlaxoSmithKline(GSK)2606414 is the first-generation PERK inhibitor that is characterized by 30 nM IC_50_ as well as good cellular in vitro and in vivo potency. It has been regarded as highly-selective for PERK, as its selectivity is >385-fold for PERK over other eIF2α kinases [259]. The transient repression of protein synthesis in a mouse model of prion disease induced in the PERK-dependent manner was challenged after treatment with small-molecule PERK inhibitor GSK2606414. Moreover, GSK2606414-treated prion-infected mice demonstrated a significantly decreased level of the phosphorylated form of PERK and eIF2α in comparison with vehicle-treated mice. Additionally, in an animal model of PrP disease, after GSK2606414 treatment, the levels of ATF4 and CHOP were markedly reduced, which confirmed the neuroprotective effect of GSK2606414 [254]. Radford et al. showed that PERK-eIF2α-dependent repression of global protein synthesis also acts as a key mediator in neuronal loss in mouse model of FTD, in which ER stress is directly evoked by mutant protein tau. Brain tissue of rTg4510 mice that overexpressed the P301L tau mutation was characterized by high level of p-PERK and p-eIF2α and transient translational repression by mice aged 6 months. The level of p-PERK, p-eIF2α, and ATF4 was significantly reduced in P301L transgene-expressing mice orally treated with GSK2606414. Furthermore, global protein synthesis was restored in GSK2606414-treated tauP301L+ mice in comparison with vehicle-treated mice. Treatment with GSK2606414 also evoked reduction of the level of phospho-tau protein, which provides the additional mechanism of protection against neurodegeneration. The histological features of the neuroprotective effect of GSK2606414 included rescue in number of cornu ammonis 1 CA1 neurons to approximately 60%, as compared to vehicle-treated mice that demonstrated profound hippocampal neuronal loss characteristic for FTD, with about 25% neurons preserved. Moreover, a significant reduction in brain atrophy as well as greater total brain weights were observed in PERK inhibitor-treated animals in comparison with untreated transgene-expressing animals at the same stage of the disease. Moreover, mentioned features after treatment with GSK2606414 were comparable to these observed in mice at early stages of the disease. Of note were that the treatment of mice started at 6 months old, when the progressive changes in the hippocampus had already begun, which supports the fact that GSK2606414 deterred already progressing neurodegeneration [260]. 

It has been reported that use of PERK inhibitor GSK2606414 may also constitute a promising treatment strategy against the neurodegeneration in PD. Interestingly, pharmacological induction of ER stress conditions by tunicamycin and oral administration of GSK2606414 triggered significant inhibition of the PERK-mediated UPR signaling pathways in the SNpc of mice injected with neurotoxin 6-hydroxydopamine (6-OHDA). Moreover, GSK2606414 treatment evoked an increased level of dopamine as well as expression of synaptic proteins [231].

It is worth mentioning that not only does GSK2606414 prevent neuronal loss, but it also positively influences cognitive function and alleviates the clinical symptoms of neurodegeneration. For instance, in the mentioned experiment by Radford et al., GSK2606414-treated mice at 8 months of age exhibited normal grooming, posture, and movement, whereas vehicle-treated animals demonstrated poor grooming, hunched posture, and impaired mobility at the same stage [260]. In another study by Ounallah-Saad et al., it has been proven that GSK2606414 is responsible for cognitive enhancement via reduction of p-eIF2α levels. After injection of the inhibitor into the rat insular cortex, the incidental taste learning and conditioned taste aversion (CTA) procedures were performed. PERK inhibitor enhanced novel taste learning, associative memory (higher aversion), as well as extinction of CTA compared to the vehicle-injected group, which suggests increase in cortical-dependent behavioral plasticity. Interestingly, genetic silencing of PERK in tested animals has also been performed with similar results [261]. A recent study by Zhu et al. has also found that inhibition of PERK with GSK2606414 affects working memory, as mice treated with the inhibitor appeared to be impaired in a spontaneous alternation Y-maze task as well as in fear extinction, which implies the impairment of spatial working memory and memory flexibility, respectively [262]. Lastly, Sharma et al. reported that reduction of PERK expression by GSK2606414 improved hippocampal-dependent memory and learning, as well as increasing neuronal excitability after injection into the CA1 region in middle-aged mice. Notably, the hippocampal memory in mice was restored to the normal performance levels observed in young individuals [263].

Interestingly, Grande et al. have demonstrated in an in vivo experimental model that inhibition of the PERK-mediated UPR response signaling branches via GSK2606414 delayed neurodegeneration, prolonged the asymptomatic phase of the disease, and markedly improved motor function in a mouse model of Marinesco–Sjögren syndrome [264].

Although there is a significant neuroprotective effect of GSK2606414 in multiple neurodegenerative disorders, it has also been reported that treatment with GSK2606414 evokes a cytotoxic effect, resulting in body weight loss and, due to increased blood glucose level, hyperglycemia in a mouse model of prion disease [254]. Moreover, treatment with GSK2606414 of prion-infected mice evoked side effects that may be correlated with pancreatic toxicity [231].

### 6.2. GSK2656157

Due to the above-mentioned side effects triggered by GSK2606414, the second generation of PERK inhibitors, which embrace GSK2656157, has been discovered. GSK2656157 is non-toxic due to better pharmacokinetics and physical properties in comparison with GSK2606414 [265]. Importantly, GSK2656157 has an IC_50_ of 0.9 nM and it is characterized by high selectivity for PERK. It has been reported that GSK2656157 may inhibit PERK activity in cells with an IC_50_ in the range of 10-30nM. Atkins et al. showed significant inhibition of PERK and eIF2α phosphorylation that was correlated with decrease in ATF4 and CHOP protein levels in a broad range of cell lines with evoked ER stress conditions [266].

Nevertheless, the latest study by Rojas-Rivera et al. has provided a solid evidence that both mentioned GSK inhibitors appear to be in fact inhibitors of the receptor-interacting serine/threonine-protein kinase 1 (RIPK1), rather than of PERK. It has been proven that both compounds effectively inhibit tumor necrosis factor (TNF)-mediated RIPK1-dependent cell death via apoptosis or necroptosis. It is of note that this effect was independent of PERK inhibition. Both GSKs inhibited TNF-mediated RIPK1-mediated cell death at a concentration that cannot affect PERK activity within cells. Surprisingly, the inhibitory activity of GSK2656157 against RIPK1 was comparable to the activity of GSK’963, about 800-fold higher than that of NEC-1 and 200-fold higher than that of NEC-1s, all of which are widely used, specific RIPK1 inhibitors. Thus, the mentioned finding implies that all experiments performed with GSK2606414 or GSK2656157 are burdened with a high risk of errors and misinterpretation. Thus there is a requirement to use a novel AMG’44 inhibitor for PERK pharmacological inactivation rather than GSKs, as far as AMG’44 is regarded as selective for PERK and not interacting with RIPK1. Additionally, further studies need to be performed in order to determine the full potency of the GSK2656157 compound in terms of efficient inhibition of additional kinases of human kinome, despite RIPK1 and PERK [265].

### 6.3. Salubrinal

A study by Moreno et al. showed that PERK-mediated eIF2α phosphorylation and subsequent transient repression of global protein synthesis led to chronic progression of neurodegenerative diseases. Treatment of prion-infected mice with salubrinal, as a small-molecule inhibitor of the eIF2α phosphatase enzymes, demonstrated the opposite effect to previously tested PERK inhibitors [13]. Salubrinal selectively inhibited eIF2α dephosphorylation, evoked an increased level of p-eIF2α, and accelerated neurotoxicity. Moreover, it markedly reduced survival in prion-infected mice as compared to prion-infected mice untreated with salubrinal. Thus, this investigation established that the inhibition of the PERK-dependent UPR signaling pathways may constitute a novel therapeutic strategy against neurodegenerative diseases [131]. Furthermore, O’Connor et al. have demonstrated that salubrinal significantly elevated β-secretase expression and subsequently Aβ generation in primary cortical neurons. Thereby, obtained results have suggested that salubrinal may promote amyloidogenesis and increased levels of p-eIF2α, which plays a key role in the molecular mechanism directly leading to AD development and progression [47]. Conversely, Huang et al. have shown that only long-term treatment (46h) with salubrinal evoked an increased level of the p-PERK-mediated AD markers. Interestingly, short-time treatment (6h) with salubrinal had no effect on eIF2α phosphorylation, but it evoked inhibition of the nuclear factor-kappa B (NF-κB) signaling pathway that protected against Aβ-mediated neuronal apoptosis and activation of microglial in rat primary cortical neurons and mouse microglial BV-2 cells [267]. Additionally, a study by Lee et al. has demonstrated that salubrinal, as an activator of UPR signaling branches, triggered significantly raised level of BiPs, resulting in attenuation of Aβ-induced neuronal apoptosis [203]. Consistently with the above-mentioned investigations that have confirmed a neuroprotective effect of Salubrinal, research by Reijonen et al. has established reduced ER stress conditions evoked by deposition of mHtt within the ER lumen of salubrinal-treated, neuronal PC6.3 cells. The neuroprotective effect of salubrinal was evoked by increased levels of p-eIF2α and BiPs, as well as reduced cleavage of caspase-12 [236].

Additionally, treatment with salubrinal appeared to be effective in several models of PD. It has been reported that salubrinal significantly attenuated the disease symptoms in animals with α-synucleinopathy. For instance, in the A53T mutant human αS transgenic (*A53TαS)* transgenic (Tg) mice, salubrinal extended the life span, reduced ER accumulation of α-syn, and delayed the onset of motor dysfunction; however, it failed to attenuate the disease progression. Interestingly, it has been suggested that the mechanism of action of salubrinal in the mentioned model resulted from activation of CHOP, the eIF2α downstream reporter, rather than from increase in p-eIF2α levels. Remarkably, salubrinal significantly decreased microsomal accumulation of monomeric and oligomeric forms of α-syn [268], consistently with the result from another report in which treatment with salubrinal attenuated the accumulation of toxic α-syn oligomers [269]. Conversely, in an *A53TαS* AAV-rat model, salubrinal treatment did not prevent dopaminergic neurodegeneration, but it attenuated the Golgi fragmentation in remaining DA neurons, thus preventing further neuronal loss and alleviated the symptoms [268]. According to another reports, the dose of 5 mM of salubrinal partially prevented *A53T*α*S*-induced cytotoxicity in the PC12 cell line, and this effect was not enhanced by further increase in dosage of the compound. However, after the addition of 50 mM z-VAD, a pan-caspase inhibitor, the cell death was almost completely evaded [270]. Numerous studies have identified that salubrinal protected SH-SY5Y cells either from rotenone- or paraquat-induced neurotoxicity via interfering with death/survival-related signaling pathways such as ATF4 or apoptosis signal-regulating kinase 1 (ASK1), respectively [271,272,273]. Salubrinal also appeared to be effective in a mouse model of kainic acid (KA)-induced hippocampal degeneration, wherein it prevented cell death in the cornu ammonis 3 (CA3) region of the hippocampus [274].

### 6.4. ISRIB

Sidrauski et al., through the cell-based screen for small-molecule inhibitors of PERK-dependent signaling pathway, have identified the ISRIB compound with 5 nM of IC_50_ and good BBB penetration. There was no effect on PERK and eIF2α phosphorylation after treatment of ER-stressed human bone osteosarcoma epithelial cells (U2OS) with the ISRIB inhibitory compound. Unexpectedly, ISRIB inhibited expression of downstream targets of the PERK-dependent UPR signaling pathways such as ATF4, CHOP, and GADD34. Furthermore, there was no inhibitory effect of ISRIB on global protein translation. Moreover, upon activation of GCN2 and HRI kinases, ATF4 expression was inhibited after ISRIB treatment. Hence, these data indicate that ISRIB acts as an inhibitor of downstream targets of all eIF2α kinases. In vivo investigation has demonstrated no cytotoxic effect of ISRIB, as well as selectively enhanced long-term memory in eIF2α+/S51A ISRIB-treated mice [275]. Importantly, ISRIB exhibited high selectivity to ER-stressed cells. Treatment with ISRIB attenuated expression of p-eIF2α-dependent targets including ATF4, CHOP, and GADD34. However, there was no significant translational changes, with the exception of a decreased level of translation of a few mRNAs including ATF4, in non-stressed cells [276]. Halliday et al. demonstrated that both GSK2606414 and ISRIB are characterized by a neuroprotective effect. After treatment with ISRIB of prion-infected mice, decreased loss of hippocampal neurons and reduced prion spongiform pathology were observed. However, in comparison with ISRIB, chronic treatment with GSK2606414, due to its high toxicity, resulted in approximately 50% reduction in pancreatic weight and destruction of pancreatic tissue in prion-infected mice. ISRIB only partially restores global protein translation, as compared with GSK2606414, which evokes complete recovery of global protein synthesis in pancreatic cells [277]. In PC12 neuronal cells treated with ISRIB, the Aβ_1–42_-induced apoptotic cell death was significantly decreased in comparison with PC12 cells treated solely with Aβ_1–42_ peptide. Moreover, ISRIB evoked inhibition of Aβ_25–35-_mediated *ATF4* expression in the PC12 cell line. However, ISRIB has no impact on Aβ generation, as Aβ production was not inhibited in ISRIB-treated HEK293T cells [278]. Although there is a significant neuroprotective effect of ISRIB without a cytotoxic effect, ISRIB cannot constitute novel UPR inhibitor against human neurodegenerative diseases due to its high insolubility [149,279]. 

### 6.5. Guanabenz

Guanabenz (GBZ) is a small-molecule inhibitor that enhances PERK-mediated UPR signaling pathways through selective inhibition of GADD34-dependent dephosphorylation of p-eIF2α. GBZ interferes with GADD34, which consequently prevents GADD34-PP1C complex assembly, resulting in an increased level of p-eIF2α and its downstream targets. GBZ also restores global protein translation, and thereby significantly increases chaperone synthesis. The aforementioned GBZ-mediated events directly evoke a prolonged pro-adaptive response under ER stress conditions in human cells [280]. A study by Wang et al. demonstrated that GBZ markedly inhibited GADD34-dependent eIF2α dephosphorylation, resulting in significant amelioration of disease onset, prolonged early phase of disease, and survival in a G93A transgenic mouse model of amyotrophic lateral sclerosis (ALS), as compared to G93A mice untreated with GBZ [281]. Contrarily, it has been demonstrated that GBZ is characterized by high affinity for α2-adrenergic receptors and antihypertensive action. However, GBZ elicits other side effects including drowsiness, dry mouth, weakness, and tiredness in patients, and thereby it cannot be used as a selective inhibitor of GADD34–PP1C assembly [282,283].

### 6.6. Sepin1

There is a plethora of studies that indicate that targeting phosphatases via small-molecule inhibitors may contribute to significant amelioration of neurodegenerative disease symptoms. Hence, Sephin1 has been extensively studied as the second selective inhibitor of holophosphatase. Sephin1, like GBZ, possesses the ability to create a complex with a regulatory subunit of PP1, namely, GADD34. This event results in prolonged eIF2α phosphorylation, under ER stress conditions, and inhibition of global protein translation recovery. Das et al. have reported that there was no alteration in the level of ATF4 in Sephin1-treated cells, but the level of pro-apoptotic CHOP protein was significantly decreased, preventing apoptotic cell death. Importantly, in comparison with GBZ, Sephin1 did not have α2-adrenergic activity. There were also no adverse effects on general health and memory observed in Sephin1-treated mice. Moreover, Sephin1 prevented motor neuron deficits and myelin thickness around axons in sciatic nerves in the mouse model (MPZ^mutant^) of demyelinating neuropathy Charcot–Marie–Tooth 1B. Moreover, loss of motor neurons was inhibited in transgenic mice expressing the human ALS-causing mutant SOD1G93A (SOD1^mutant^), treated with Sephin1 [284]. Recently, this above-mentioned molecular mechanism, inhibited upon treatment of cells with activated ER stress conditions with Sephin1, was questioned by Crespillo-Casado et al. Surprisingly, investigation by Crespillo-Casado and colleagues demonstrated that Sephin1’s ability to suppress neurodegeneration was not due to the inhibition of eIF2α dephosphorylation via abrogation of GADD34-PP1C complex assembly. Thus, additional studies are necessary to discover alternative mechanisms that are responsible for Sephin1-mediated suppression of neurodegeneration [285].

### 6.7. Trazodone Hydrochloride and Dibenzoylmethane

The National Institute of Neurological Disorders and Stroke (NINDS) small-molecule library screening has identified two compounds with neuroprotective activity: trazodone hydrochloride and dibenzoylmethane (DBM). Trazodone is a well-known multi-target drug that belongs to the class of both serotonin receptor antagonists and reuptake inhibitors (SARIs) and serotonin type 2 (5-HT_2A_ and 5-HT_2C_) receptor antagonists [286]. Due to its dual mechanism of action, it is capable of overcoming the adverse effects and tolerability issues associated with selective serotonin reuptake inhibitors (SSRIs) or serotonin–norepinephrine reuptake inhibitor (SNRI) therapy. In addition, trazodone exerts an antagonistic effect against histamine-H1, α1-adrenergic and α2-adrenergic receptors, as well as minimal anticholinergic activity [287]. For this reason, it has been widely used not only as an antidepressant, but also in treatment of numerous medical conditions including insomnia, posttraumatic stress disorder, obsessive compulsive or anxiety disorders, feeding and eating disorders, substance abuse, behavioral disturbances associated with cognitive dysfunction, fibromyalgia, and neuropathic pain [288]. A study by Halliday et al. demonstrated that both of the mentioned compounds possess the ability to significantly inhibit the p-eIF2α-dependent repression of global protein synthesis. Treatment with trazodone hydrochloride and DBM prion-infected mice prevented loss of hippocampal neurons, restored memory deficits, abrogated development of neurological signs, and markedly prolonged mice survival. Moreover, in an animal model of tauopathy-FTD, the tested small-molecule compounds rescued memory deficits and significantly reduced loss of hippocampal neurons. Importantly, both compounds are non-toxic to the pancreas and are characterized by good pharmacokinetic properties [279].

### 6.8. LDN-0060609

The newest data have reported that the small-molecule inhibitor of the PERK-dependent signaling pathway LDN-0060609, selected from the Laboratory for Drug Discovery in Neurodegeneration (LDDN) compound library, may constitute a novel, ground-breaking treatment strategy against neurodegenerative diseases, especially AD. It has been demonstrated that LDN-0060609 is non-cytotoxic, has no effect on cell cycle progression, and evokes a significant inhibition of the eIF2α phosphorylation in the phenotype of type 1 rat normal astrocytes derived from diencephalon (DI TNC1) cell line. Thereby, LDN-0060609 significantly attenuates the pro-apoptotic, PERK-mediated signalling pathway and enhances cell survival in vitro; thus, it may contribute to prevention of apoptosis and the resulting neurodegeneration in terms of AD. However, the actual mechanism of action of the inhibitor is yet to be identified. It would also be intriguing to investigate whether this mentioned compound in fact exerts neuroprotective effects in animal models of not only AD but also other neurodegenerative diseases, including PD, ALS, and prion disease [289].

### 6.9. Oxyresveratrol

Resveratrol with its two analogues, oxyresveratrol (OXY) and piceatannol (PINO), are polyphenolic stilbenes derived from mulberry (*Morus alba*) fruits, already known to exert antioxidant and neuroprotective effects [290]. Among all three compounds, PINO has been confirmed to have the strongest anti-amyloidogenic properties in an AD in vitro model, wherein it decrease Aβ levels via activation of α-secretase and MMP-9, without inducing cell death [291]. Moreover, recently a novel, self-microemulsifying formulation of OXY appeared to be effective against Aβ aggregation in an AD murine model, as well as resulting in a significant dose reduction due to higher bioavailability [292]. In PC12 cells, a common model for both AD and PD, PINO inhibited Aβ-induced ROS generation and apoptosis, α-syn fibril formation, and α-syn-induced cytotoxicity [293,294]. Interestingly, a recent study by Shah et al. revealed that the underlying mechanism of action of OXY is strictly correlated with the PERK-dependent UPR signaling pathway. In 6-OHDA-exposed Mes23.5 cells, one of the in vitro PD models, treatment with OXY significantly prevented the transcription of ATF4 with its main downstream target, CHOP, even at the lowest concentration of the drug. In the second cellular model of PD, SH-SY5Y cells overexpressing A30P mutant α-syn, OXY restored the increased level of another UPR-related protein, GRP78. However, the direct impact of OXY on CHOP-associated apoptosis as well as on the other branches of the UPR signaling pathway needs to be further elucidated [295].

### 6.10. β-Asarone

Numerous in vivo studies have confirmed the positive effects of application of asarone derivatives, such as α- or β-asarone, on cognitive function and alleviation of AD symptoms in animal models [296,297,298]. Anti-amyloidogenic properties of mentioned compounds have also been observed in the PC12 cell line, and likewise an anti-neuroinflammatory effect in BV2 microglial cells [299]. Recently, it has been discovered that β-asarone improved behavioral functioning of 6-OHDA-induced parkinsonian rats. β-Asarone inhibited the expression of GRP78, CHOP, p-IRE1α, and XBP1 within neurons, suggesting that its possible mechanism of action depends on two major UPR-related pathways, PERK- and IRE1α-dependent pathways [300]. Recent investigation on the same animal model have revealed that treatment of rats with β-asarone inhibited the PERK/CHOP/Bcl-2/Beclin-1 axis with simultaneous downregulation of GRP78 and upregulation of Bcl-2. These results imply the key role of β-asarone in regulation of ER stress-associated autophagy, and they were consistent with the ones obtained in the PERK inhibitor-treated group [301].

### 6.11. Gastrodia Elata Derivatives

**Figure 2:**
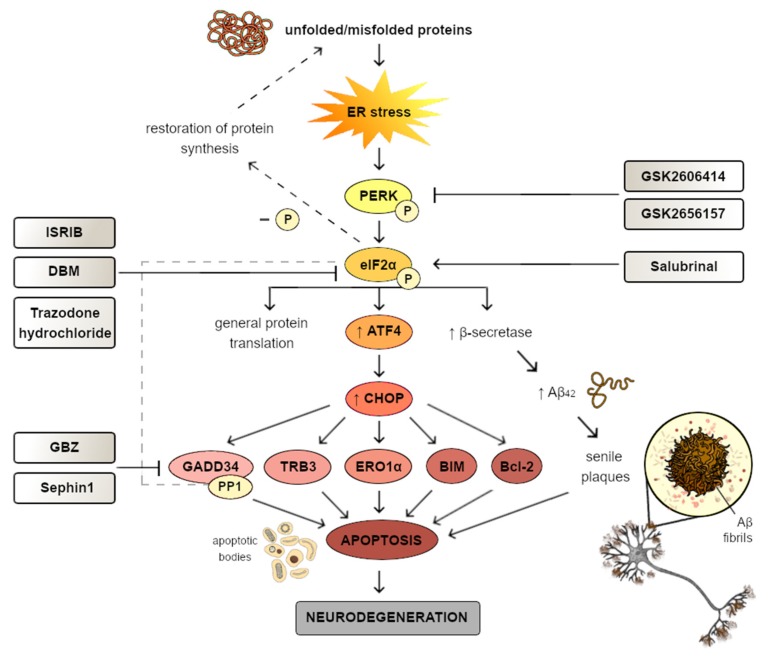
Small-molecule inhibitors of the protein kinase RNA-like endoplasmic reticulum kinase (PERK)-dependent UPR signaling pathway and their targets of action. Numerous studies have suggested that the pathogenesis of neurodegenerative diseases, including Alzheimer’s disease (AD), is correlated with the accumulation of misfolded and unfolded proteins, which may directly or indirectly trigger endoplasmic reticulum (ER) stress conditions and activation of the PERK-dependent unfolded protein response (UPR) signaling pathways. The upregulation of specific mechanisms related to the PERK branch of UPR signaling and AD pathogenesis is indicated by arrow, downregulation by inhibiting line, whereas GADD34-mediated dephosphorylation of eIF2α in a negative feedback loop by dotted arrow. Under mild-to-moderate ER stress conditions, UPR has a pro-adaptive role, whereas severe or long-termed ER stress conditions directly evoke shift of the UPR toward its pro-apoptotic branch, leading to neurodegeneration. The newest data have reported that pharmacological modulation of the PERK-dependent branch of the UPR signaling pathway via small-molecule inhibitors may constitute a novel treatment strategy against neurodegeneration.

Numerous compounds have been extracted from *Gastrodia elata* Blume (GE), a traditional herbal medicine, and their antioxidant properties have been under detailed investigation. Gastrodin (Gas) has been found to suppress BACE1 expression in Tg2576 mice and H_2_O_2_-stimulated SH-SY5Y cells via inhibition of PKR and eIF2α activation. The downregulation of BACE1 resulted in improvement in learning and memory abilities in treated mice as well as it attenuated oxidative stress in hippocampus on the molecular level. However, it has been suggested that Gas-induced inhibition of eIF2α phosphorylation was independent from PERK activation [302]. In another study, GE and its two components, Gas and 4-hydroxybenzyl alcohol (4HBA), were shown to prevent BV2 mouse microglial cells from Aβ-induced cell death via inhibition of CHOP-mediated branch with simultaneous upregulation of GRP78 chaperones [303]. On the contrary, bibenzyl compound 20C, a novel compound isolated from GE, has been demonstrated to protect PC12 cells against tunicamycin-induced ER stress. Co-treatment of cells with 20C induced suppression of all three UPR-related signaling branches, including the PERK-dependent one. It has been demonstrated that the levels of p-eIF2α and ATF4 were significantly decreased. Moreover, 20C reduced the accumulation of α-syn within cells concurrently, which further improved the cell viability [304]. In a separate experiment, it was found that 20C attenuated apoptosis and oxidative stress in rotenone-treated PC12 cells via activation of the nuclear factor erythroid 2-related factor 2 (Nrf2)/antioxidant responsive element (ARE)/heme oxygenase-1 (HO-1) signaling pathway, which is strictly correlated with the PERK-mediated branch of the UPR signaling pathway [305]. With regard to all these aspects, the actual mechanism of action of GE and its derivatives is yet to be confirmed (Figure 2).

## 7. Summary and Perspective

As previously indicated, the pathogenesis of a broad range of neurodegenerative diseases in most cases may be correlated with common molecular process, namely, aggregation of misfolded and unfolded proteins within the nervous tissue. Nevertheless, the role of the PERK-dependent UPR signaling branch in the process of neurodegeneration still remains controversial. There are many contradictory results described in the literature, even in cases where researchers referred to similar issues or the studies were conducted on the same disease models. It needs to be clarified whether the ER stress conditions and subsequent activation of the PERK branch are evoked by accumulation of protein aggregates within neurons (tau, α-syn, mHtt, PrP^Sc^) or surrounding nervous tissue (Aβ, PrP^Sc^), as one report may indicate that they indeed are activated, whereas others state that they are not. To fully address this issue, it is recommended that the studies be repeated with particular emphasis on the improvement of currently used experimental methods. Another problem to be resolved appears to be the mechanism in which disturbances in the ER, as reported in numerous proteopathies, are actually induced, considering the fact that most of mentioned proteins are not strictly associated with the ER lumen and they rather accumulate in the cytoplasm or extracellularly. Although the molecular mechanism that evokes shift of the UPR from pro-adaptive toward pro-apoptotic still remains unclear, there is a plethora of studies that have demonstrated that inhibition of the PERK-dependent UPR signaling pathways may constitute a novel, ground-breaking treatment strategy against neurodegenerative diseases. Numerous studies have confirmed the neuroprotective effect of targeting this particular branch in various types and models of neurodegenerative disorders. However, there is still missing data on many aspects of such therapeutic interventions, as well as lack of in vivo studies, as all the potential adverse effects should be taken into consideration. In addition, the actual mechanism of action of numerous potential PERK inhibitors also needs to be clarified, as their impact on the other two branches of the UPR as well as crosstalk between them and other signaling pathways remain elusive and not fully understood. Moreover, the results of studies on neurodegeneration that required the use of GSK inhibitors may be in fact irrelevant, as it has been proven that both inhibitors are more prone to inhibit the RIPK1-dependent apoptotic pathway rather than PERK. It should also be kept in mind that the mechanism of action of currently used PERK inhibitors is often based on selective inhibition of eIF2α phosphorylation, which constitutes the target of other kinases as well. Therefore, it is highly necessary to perform research on recently developed inhibitors such as AMG’44, which are both highly selective for PERK and their potential has not been fully elucidated in terms of neurodegenerative diseases. To sum up, further investigation needs to be done with regards to all mentioned issues. A thorough understanding of molecular processes involved in the pathomechanism of neurodegeneration is the key to develop an optimized, targeted therapeutic approach against all the mentioned (yet) incurable diseases.

## Figures and Tables

**Table 1:** The correlation between the expression of PERK-dependent unfolded protein response (UPR)-related proteins and proteostasis disturbances in various neurodegenerative disorders. The upregulation of specific UPR markers in the listed experimental models is indicated as an up-arrow (↑), whereas downregulation as a down-arrow (↓).

Neurodegenerative Disease	UPR Markers	Experimental Model
**Alzheimer’s disease**	↑ GRP78	Aβ-treated SK-N-SH cells [203], RBE4 cells [204], Aβ_1–40_-treated RBE4 cells [205], Aβ_1–42_-treated bEnd.3 cells [206], 5XFAD mouse model [207], APP/PS1 transgenic mice [208], Aβ_1–42_-treated rat astrocytes, 3xTg-AD mice [209], mutant PS1 cells, 3xTg-AD mice [210].
	↓ GRP78	PS1 mutant SK-N-SH cells [211].
	↑ PERK	Hippocampus of human AD brains [48], 5XFAD mouse model [160,166], Aβ-treated SK-N-SH cells [203], JNPL3 mice, rat cortical neurons [212], Aβ_42_ transgenic flies [213], pR5 mice [214].
	↑ p-eIF2α	AD mouse model [215], hippocampus of human AD brains [48], 5XFAD mouse model [160,166], APP/PS1 mice [216], Aβ-treated SK-N-SH cells [203], JNPL3 mice, primary cultures of rat cortical neurons [212], Aβ_1–42_-treated rat embryonic hippocampal neurons [217], pR5 mice [214], Aβ_1–42_-treated rat primary cortical neurons [218], Aβ_1–42_-treated rat cerebral cortical astrocytes [209].
	↑ ATF4	5XFAD mouse model [160,166], Aβ_1–40_-treated RBE4 cells [205], human *APOEe4* allele-expressing human and mouse AD models [219], Aβ_1–42_-treated rat embryonic hippocampal neurons, Aβ_1–42_-treated mice, human AD brains [217], Arg-61 APOE astrocytes [220].
	↑ CHOP	Aβ-treated SK-N-SH cells [203], primary rat cortical neurons [212], RBE4 cells [204], temporal cortex of human AD brains [221], Aβ_1–40_-treated RBE4 cell line [205], Aβ_1–42_-treated bEnd.3 cells [206], 5XFAD mouse model [207], APP/PS1 transgenic mice [208].
	↑ GADD34	J20 mice [222], AD mouse model, human AD brains [223].
**Parkinson’s disease**	↑ GRP78	SYN120 mice, SH-SY5Y+ cells, HEK 293 cells [95], PARK14 knock-in mouse model [224], 6-OHDA-treated MN9D cells [225], 6-OHDA-treated SH-SY5Y cells [226].
	↓ GRP78	Rat PD model [227,228], human PD brains [229], *DJ-1* KO neurons, MEFs and KD SH-SY5Y+ cells [230].
	↑ PERK	Human PD brains, rat PD models [231], PARK20 fibroblasts [232], mouse model of chronic MPTP/P injection [233], rat PD model [228], PARK14 mice [224], 6-OHDA- or MPP^+^-treated MN9D cells [225], *Drosophila PINK1* and *PARKIN* mutants [234], *DJ-1* KO MEFs [230].
	↑ eIF2α	PARK20 fibroblasts [232], mouse model of chronic MPTP/P injection [233], 6-OHDA- or MPP^+^-treated MN9D cells [225], *DJ-1* KO MEFs [230].
	↑ ATF4	Rat PD model [235], PD cellular models [95], mouse model of chronic MPTP/P injection [233], PARK20 fibroblasts [232].
	↓ ATF4	*DJ-1* KO neurons, MEFs and KD SH-SY5Y+ cells [230].
	↑ CHOP	6-OHDA- or MPP^+^- treated MN9D cells [225], 6-OHDA-treated SH-SY5Y cells [226], PARK14 mice [224], rat PD model [228], PARK20 fibroblasts [232].
	↓ CHOP	*DJ-1* KO neurons, MEFs and KD SH-SY5Y+ cells [230].
**Huntington’s disease**	↑ GRP78	PC6.3 cell [236], mHtt-expressing 293 Tet-Off cells [237], PC12-Q79 cells [238], HEK293T cells [239], Htt150Q-expressing N2a cells [240].
	↓ GRP78	mHtt-expressing mouse striatal cell lines [241].
	↑ PERK	Human and murine striatal cells, N171-82Q mice [242], PC12-Q79 cells [238].
	↑ eIF2α	Human and murine striatal cells, N171-82Q mice [242], 120Q-Htt-expressing PC6.3 cells [243].
	↑ ATF4	Q7 cells [244].
	↓ ATF4	Q111 cells [244].
	↑ CHOP	PC6.3 cell [236], PC12-Q79 cells [238], Q7 and Q111 cells [245], HEK293T cells [239].
**Prion disease**	↑ GRP78	PrP-expressing N2a cells [246], PrP(106-126)-treated NT2 rho0 cells and NT2 rho0+ cells [247], PrP-expressing N2a cells [248], 263K infected hamsters brain tissues, PrP-expressing 293-T cells [249], brains of BSE cattle [250], PrP-treated M17 cells [251].
	↓ GRP78	Prion infected GRP78^+/−^ and GRP78^+/+^ mice, CAD5 cell line [106], *CaBP*-KO mice [252], PrP-treated CR7 cells [253].
	↑ PERK	Prion-infected mice [254], POM1-treated cultured organotypic cerebellar slices [255], FTgpi mice [256].
	↑ p-eIF2α	Prion-diseased mice [131,254], POM1-treated cultured organotypic cerebellar slices [255], FTgpi mice [256].
	↑ ATF4	POM1-treated cultured organotypic cerebellar slices [255].
	↑ CHOP	FTgpi mice [256], PrP-expressing N2a cells [246], *CaBP*-KO mice [252], PrP-treated SH-SY5Y cells [257].
